# Supplementary figures and images for: Using population-specific add-on polymorphisms to improve genotype imputation in underrepresented populations
Source: PLoS Comput Biol. 2022 Jan 13;18(1):e1009628. doi: 10.1371/journal.pcbi.1009628 (PMC8791479; doi:10.1371/journal.pcbi.1009628)

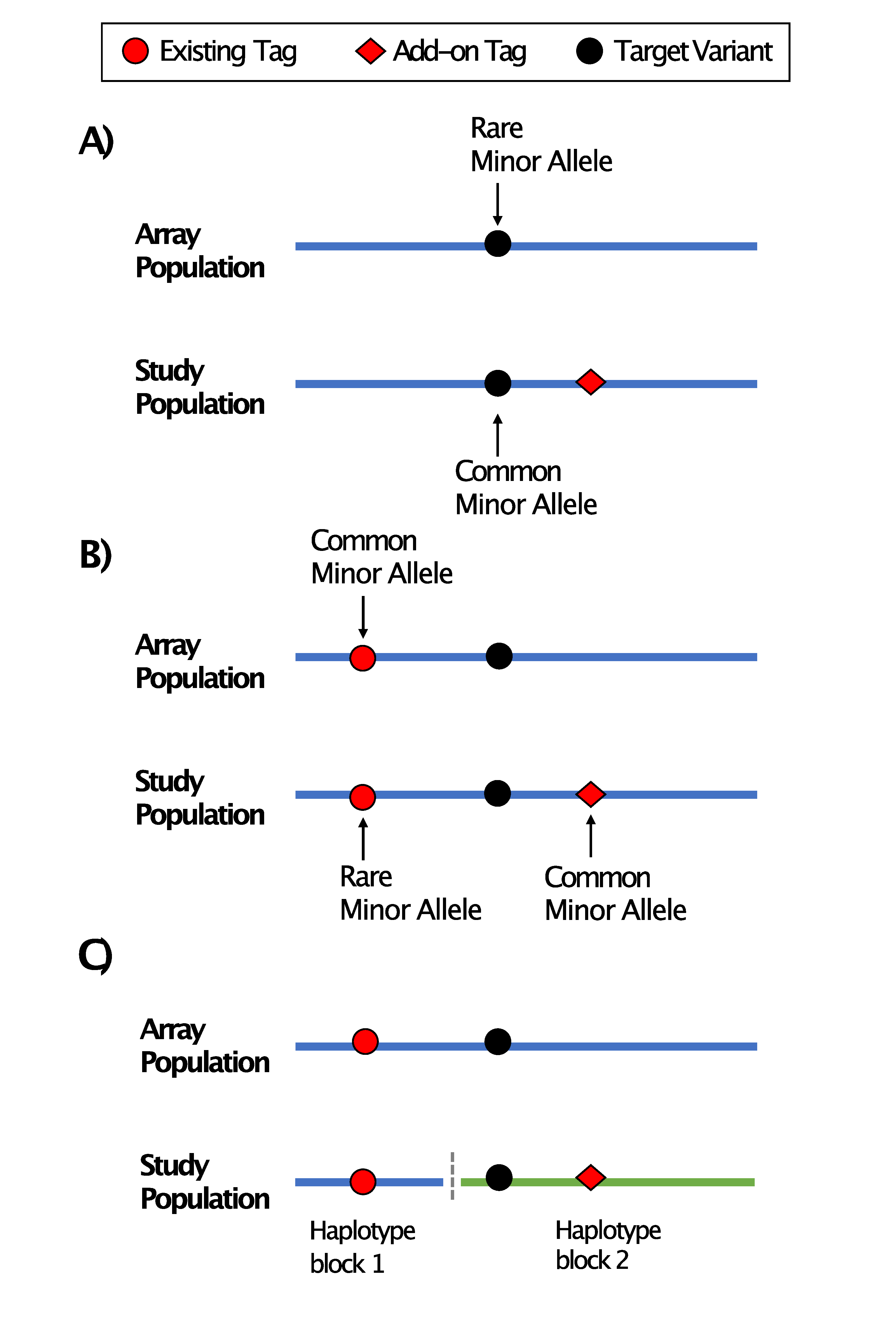

Supplement: S1 Fig — The array population represents the population that the existing genotyping array is designed for. The study population represents the population that one would like to genotype and for which the add-on tags are designed for. A) A target variant with a minor allele that is rare in the array population but common in the study population. B) An existing tag with a minor allele that is common in the array population but rare in the study population. C) The haplotype structure is different between the array population and the study population. (TIF) [file pcbi.1009628.s002.tif]

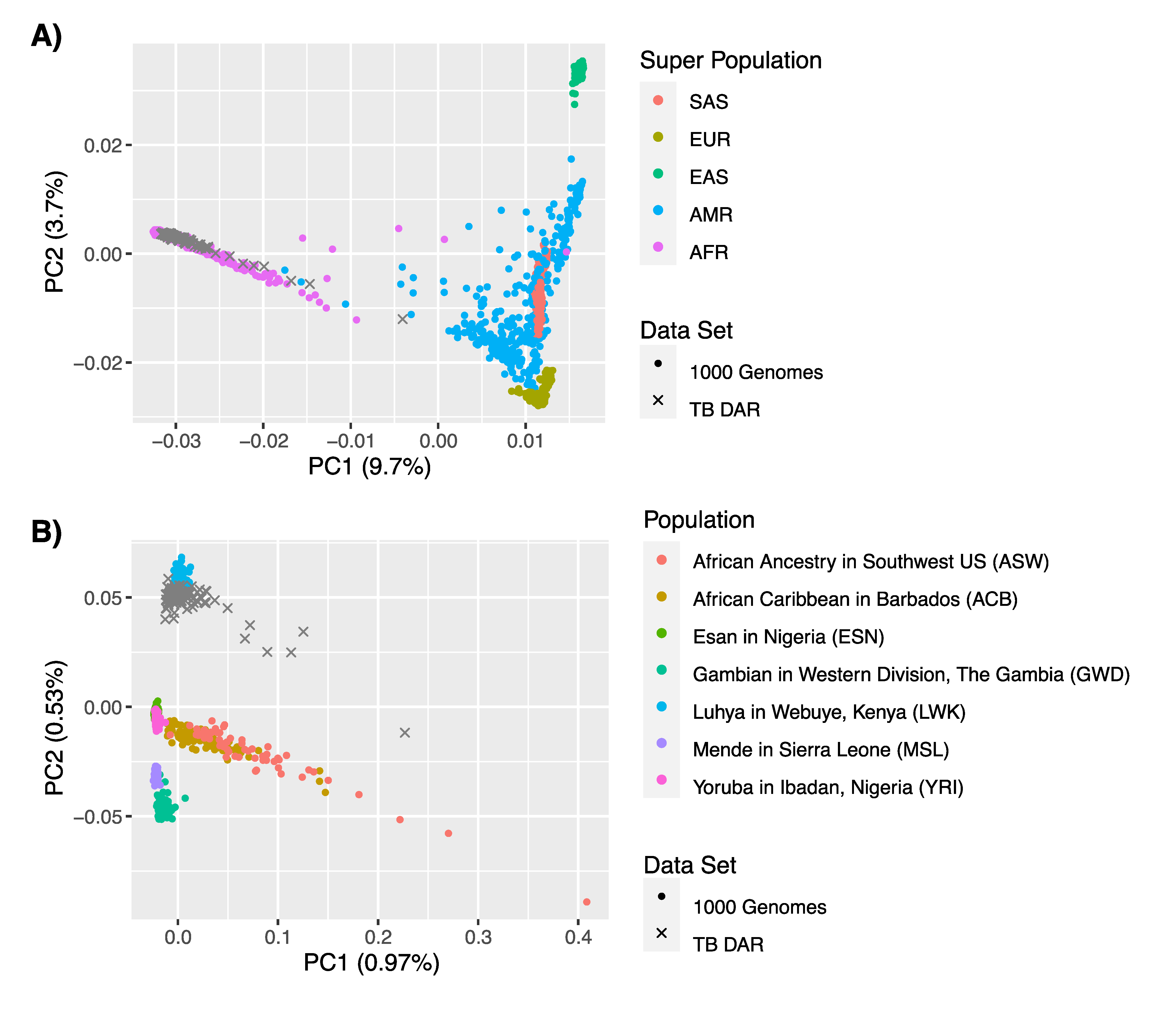

Supplement: S2 Fig — Percent of variance explained by each PC are indicated in brackets. A) All 1000 Genomes populations, grouped according to super-populations. B) 1000 Genomes African populations. (TIF) [file pcbi.1009628.s003.tif]

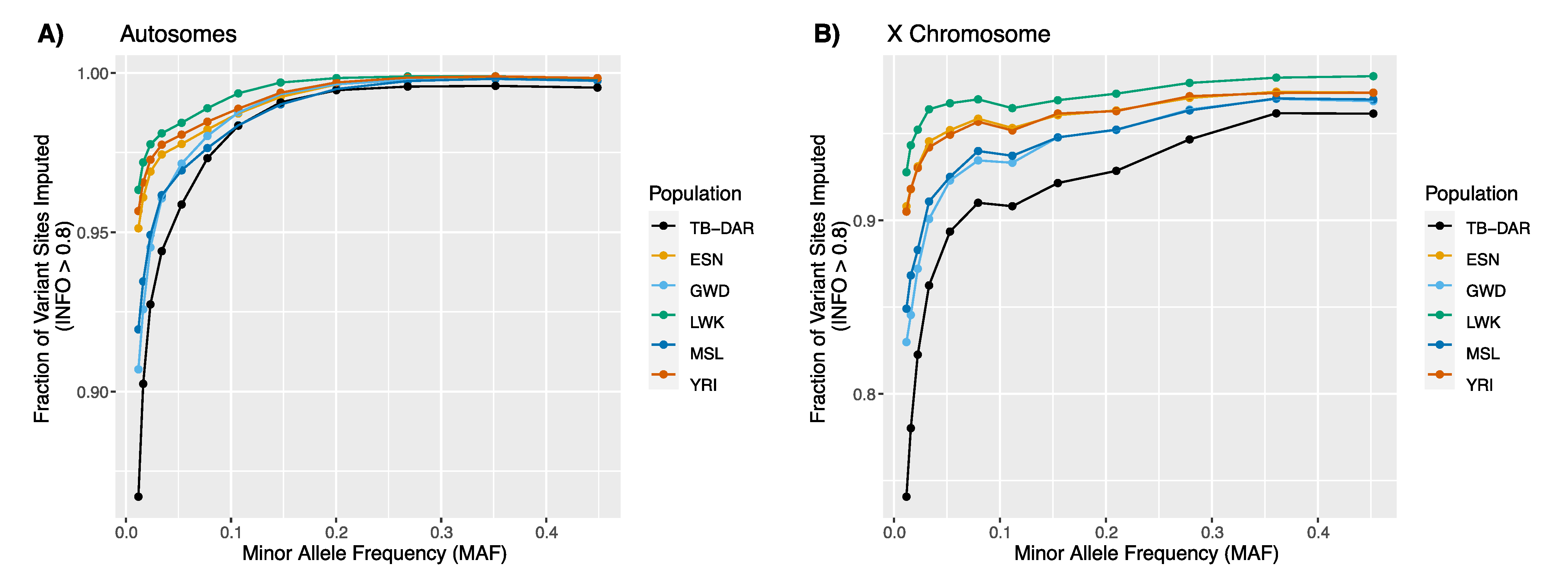

Supplement: S3 Fig — A) Fraction of autosomal variant sites successfully imputed (INFO > 0.8) B) Fraction of X-chromosome variant sites successfully imputed (INFO > 0.8). (TIF) [file pcbi.1009628.s004.tif]

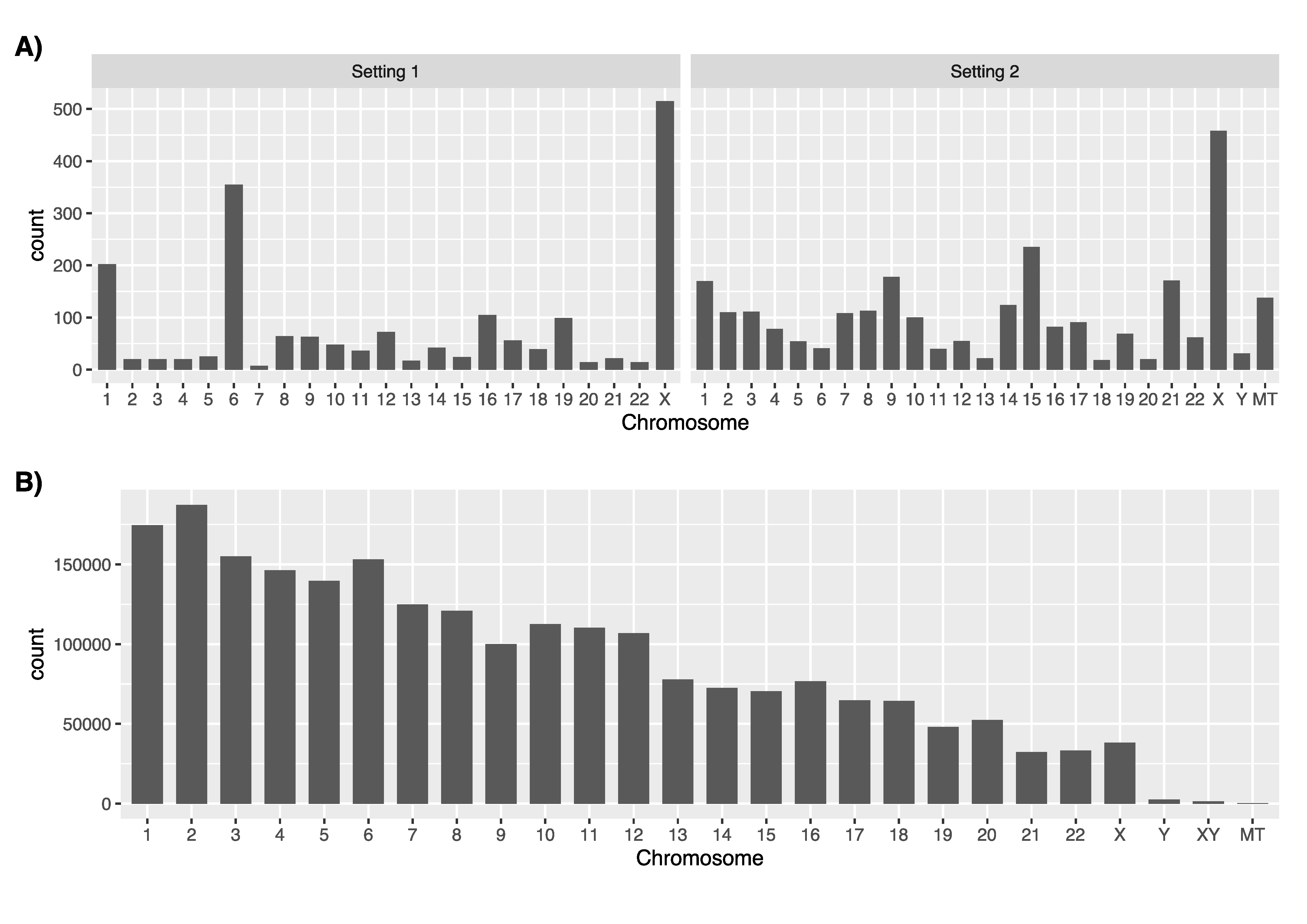

Supplement: S4 Fig — A) Add-on tags SNPs selected based on Settting 1 and Setting 2. B) Existing tags SNPs on the H3Africa array. (TIF) [file pcbi.1009628.s005.tif]

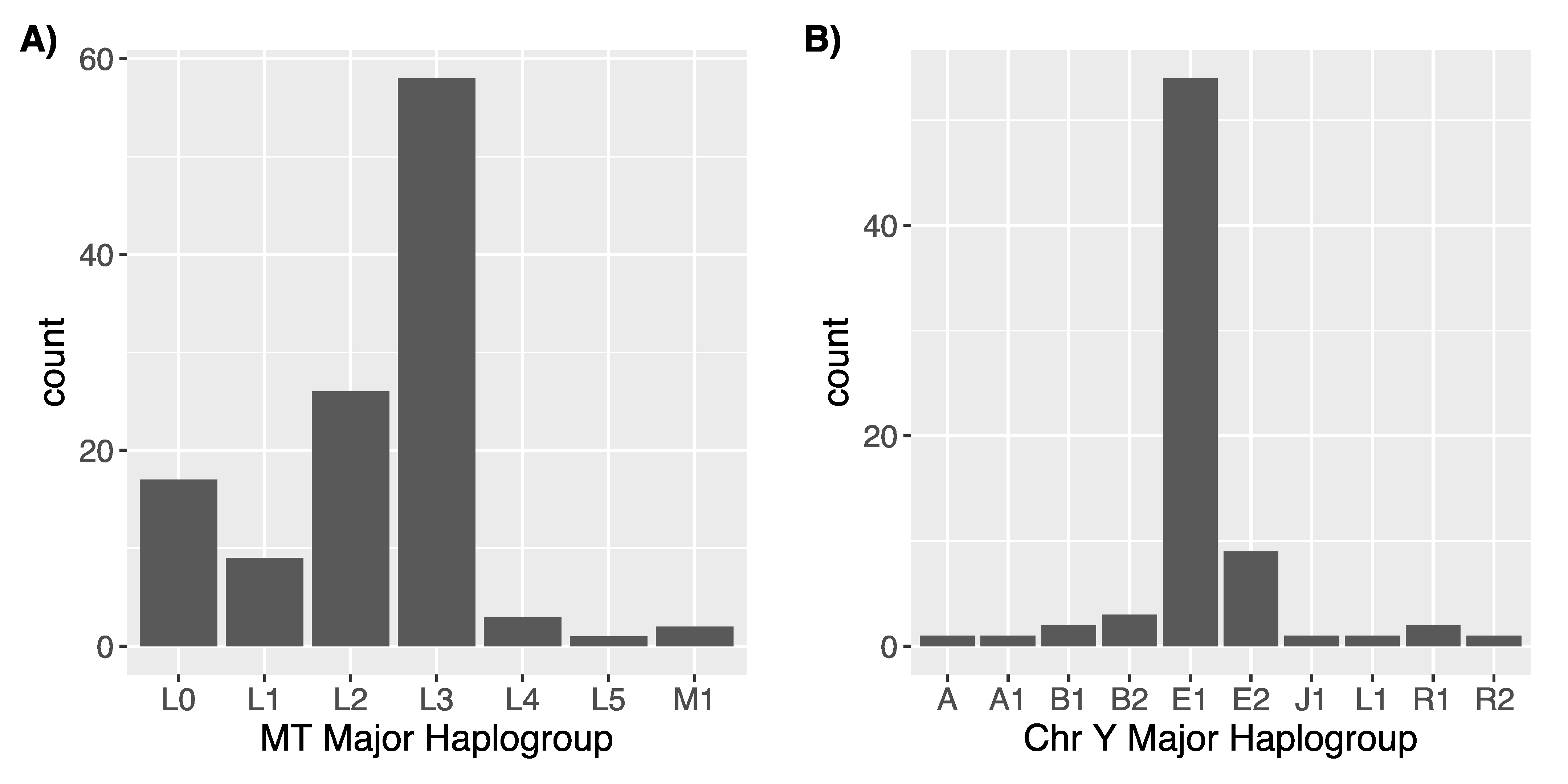

Supplement: S5 Fig — A) Mitochondria B) Y chromosome (Males Only). (TIF) [file pcbi.1009628.s006.tif]

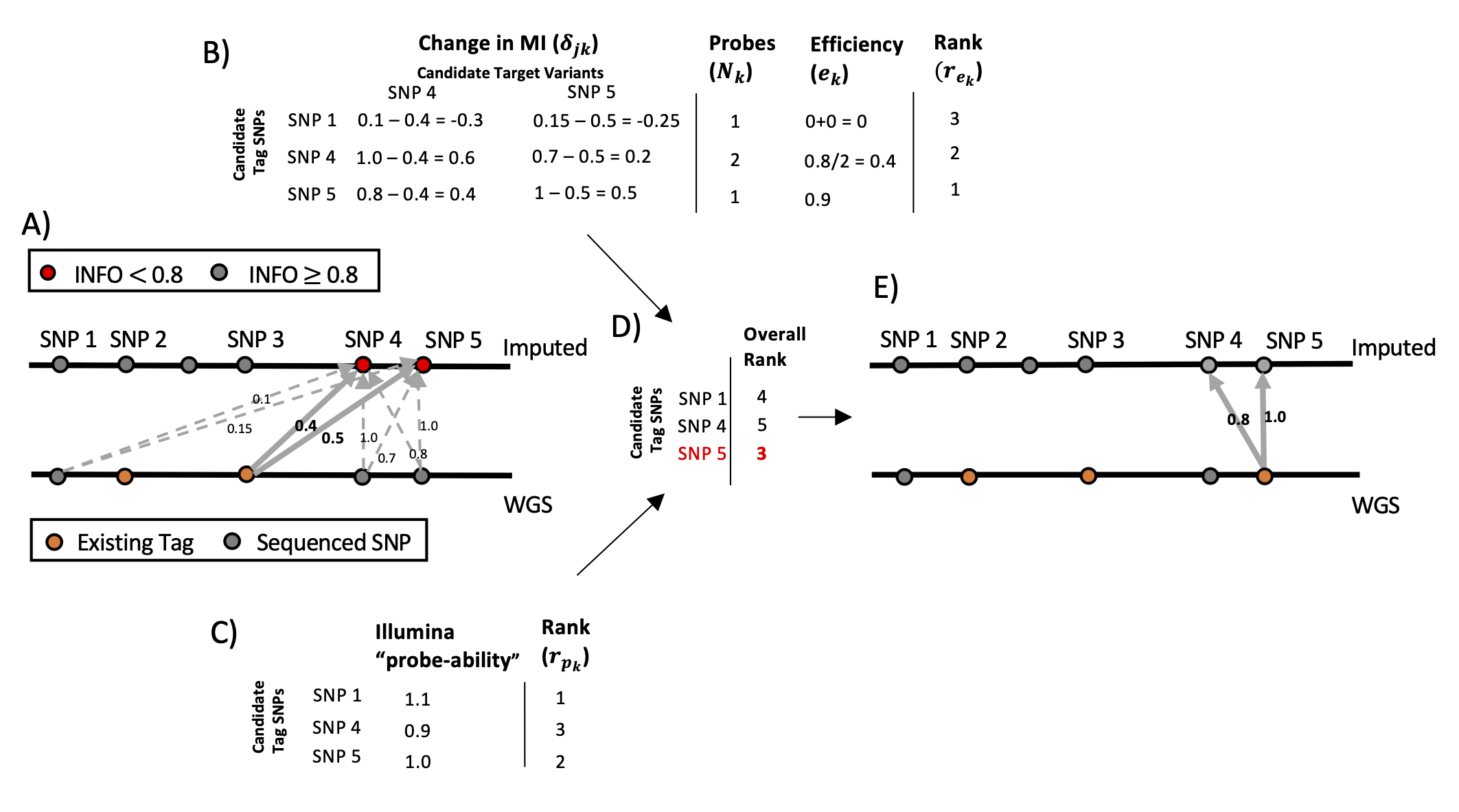

Supplement: S6 Fig — Edges between variants represent the strength of Linkage Disequilibrium (LD), measured by Mutual Information (MI). The optimal candidate tag SNP is selected based on the best overall rank, taking into account the efficiency (ek), the number of probes required (Nk), and the quality of the probe (Illumina probe-ability). In subsequent iterations, the newly added tags are incorporated as existing tags, such that the change in MI (δjk) includes the contribution of add-on tags. (TIF) [file pcbi.1009628.s007.tif]
