# Supplementary material for: Using population-specific add-on polymorphisms to improve genotype imputation in underrepresented populations
Source: PLoS Comput Biol. 2022 Jan 13;18(1):e1009628. doi: 10.1371/journal.pcbi.1009628 (PMC8791479; doi:10.1371/journal.pcbi.1009628)
Supplement: S1 Appendix — (PDF) [file pcbi.1009628.s008.pdf]

S1 Appendix

To measure the pairwise LD between a candidate tag SNP and a candidate target SNP, we utilized pairwise mutual information (MI). The pairwise MI between SNP i and SNP j (I_{ij}) was defined as:

$$I_{ij} = H_i + H_j + H_{ij}$$

where H_i denotes the entropy of a single SNP and H_{ij} denotes the joint entropy defined as:

$$H_i = -\sum_{k=0}^{2} \frac{N(g_i = k)}{N} \log_2 \left(\frac{N(g_i = k)}{N} \right)$$

$$H_{ij} = -\sum_{k=0}^{2} \sum_{k'=0}^{2} \frac{N(g_i = k, g_j = k^{'})}{N} \log_2 \left(\frac{N(g_i = k, g_j = k^{'})}{N} \right)$$

where $N(g_i = k)$ denotes the number of individuals with genotype k for SNP i, $N(g_i = k, g_j = k^{'})$ denotes number of individuals with genotype k for the SNP i and genotype $k^{'}$ for the SNP j, and N denotes the total number of individuals.

The following figure illustrates the differences between MI and r^2 (classical measure of LD). Pairwise MI was weaker than r^2 between random pairs of lower frequency SNPs ($0.05 \leq$MAF≤ 0.25), but similar to r^2 for higher frequency SNPs ($0.25 \leq$MAF≤ 0.5). This indicates that r^2 tend to overestimate the correlation between lower frequency SNPs. Thus, MI was chosen to indirectly favour the inclusion of higher frequency SNPs as add-on tag SNPs.

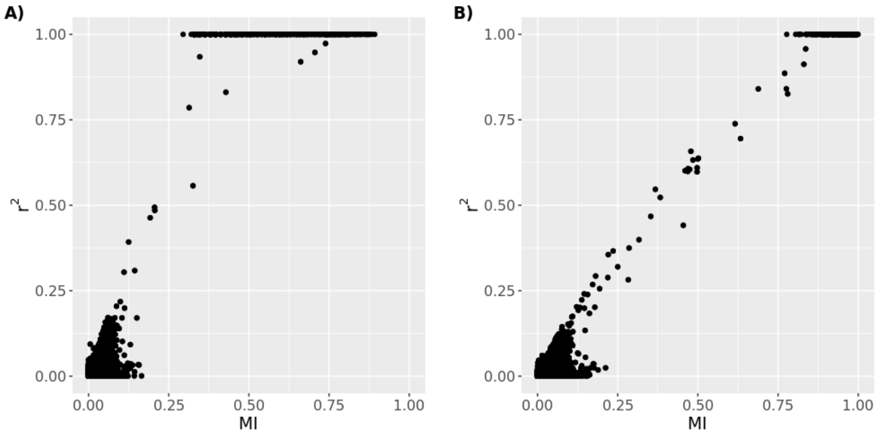

Comparison of two LD metrics: pairwise r^2 and Mutual Information (MI). LD was measured between 500 random pairs of SNPs. **A)** SNPs with low minor allele frequencies ($0.05 \leq \text{MAF} \leq 0.25$) **B)** SNPs with high minor allele frequencies ($0.25 \leq \text{MAF} \leq 0.5$).
