# Supplementary material for: Using population-specific add-on polymorphisms to improve genotype imputation in underrepresented populations
Source: PLoS Comput Biol. 2022 Jan 13;18(1):e1009628. doi: 10.1371/journal.pcbi.1009628 (PMC8791479; doi:10.1371/journal.pcbi.1009628)
Supplement: Attachment — Submitted filename: PLOS Review.pdf [file pcbi.1009628.s010.pdf]

Review Summary:

Using population-specific add-on polymorphisms to improve genotype imputation in underrepresented populations

In Genome-Wide Association Studies (GWAS), statistical inference of untyped variants in genome is needed to enhance coverage of genotyping arrays, which is referred as genotype imputation. The imputation process is performed based on external reference panels where observed correlations between variants on these panels can be indicative of untyped variants on the studied panels. The more genetically similar the reference and study populations are, the more reliable the process of genotype imputation becomes. Here, the problem lies with underrepresented populations, for whom, the reference panels on hand may not be adequately representative due to their population-specific genetic structures. In such cases, selected SNPs through genotype imputation relying on existing reference population may fall short of the actual genetic variation in the study population.

As a remedy, Whole-Genome Sequencing (WGS) can be suggested to carry out GWAS in underrepresented populations. Although, necessity of large sample sizes compromises high-priced whole-genome sequencing despite its potential. Alternately, population-specific reference panels can be constructed to improve genotype imputation. However, such efforts still remain insufficient to capture the entire genetic variability in African populations notably, due to relatively higher genetic diversity among them. Accordingly, the authors propose a cost-effective solution to the poor genotype imputation issue, that involves selection of population-specific SNPs to add to available genotyping arrays. WGS of 10% of the entire sample can benefit the inclusiveness of available reference arrays. Also, WGS of sub-sample reveals population-specific allele frequency and haplotype structure differences, that can help the selection of add-on SNPs, thus enhancing the imputation of poorly tagged SNPs in underrepresented populations. Furthermore, the approach is claimed to improve haplogroup calling in mitochondria and Y chromosome variants.

The authors showcase their approach on a Tanzanian cohort, which is not incorporated in the existing reference panels. They validate their choice of cohort by computing the genetic difference between the selected Tanzanian cohort and 1000 Genomes African population with respect to genome-wide fixation index. Here, the usefulness of the population-specific add-on SNPs for Tanzanian cohort seems justified. This comparison provides a ground to conclude that

geography play a role in genetic differentiation of African populations such that geographically closer populations have more genetic similarities, which is also supported by a genetic principal component analysis.

Selection of add-on SNPs is performed under two settings with different objectives: One guaranteeing coverage and the other, efficiency. For evaluation of the approach, imputation quality is measured on the priorly divided WGS test sample, while selected add-on SNPs under both settings are included separately while tagging target SNPs. Under both settings, an overall improvement of imputation accuracy in terms of INFO score and r^2 is obtained. The authors illustrate these findings with an exemplary region, where they observe proximity between add-on and poorly tagged target SNPs. Finally, mitochondria and Y chromosome haplogroups are analyzed in the cohort where a certain number of haplogroup marker SNPs are selected as add-ons. Here, the accuracy of haplogroup calling is increased by 22% in case of mitochondrial variants compared to the existing reference array, whereas no such improvement is gained for Y chromosome possibly due to sufficient coverage of the existing reference array. In the end, experimental results on the Tanzanian cohort affirm that consideration of poorly imputed add-on SNPs on the existing reference arrays improves genotype imputation of the common variants in the underrepresented cohort.

Comments:

First of all, I really enjoyed reading this paper. The style is easy to follow and the authors accompany the reader all the way for a better understanding of the topic.

The introduction successfully connects the sources of the problem of improving imputation to a clear statement of the purpose of this work. The potentials and drawbacks of available remedies to the issue are clearly stated which pave the way for the suitability of the suggested study. Choice of Tanzanian cohort is reasoned properly. The authors are able to voice the strengths of their approach in terms of its generalizability to any other population, its cost-effectiveness and promise in empowering GWAS. I would like to congratulate them for their work.

Some major and minor issues I might suggest revising are given below:

Major Issues:

- In the introduction, the potentials and drawbacks of available remedies to the issue are clearly stated which pave the way for the suitability of the suggested study. However, how the authors deduce insufficient coverage of African genetic variation from the references about relatively high genetic diversity of African Populations remains rather questionable and unsupported by references. It would be really nice to strengthen the motivation of the approach at this point.
- An improvement of 22% in haplogroup calling in mitochondria is stated in the final section of the results, whereas no such improvement is observed in the case of Y chromosome, which is said to be caused by lack of add-on SNPs. But the last phrase in the introduction indicates a useful selection scheme for mitochondria and Y chromosome, when in fact such a selection scheme did not work for Y chromosome. The results could be better laid out if the authors would tone down their claims for Y chromosome in the introduction.
- The first paragraph in discussion refers to previous studies implementing the idea proposed in this paper, both for Type 2 Diabetes, and states their outcomes as a supporting evidence. Moving this statement to the introduction in the related work might make more sense so that the reader is exposed to all the related work and current state-of-the-art from the beginning. The contribution of this paper on top of these previous applications should also be stated in the introduction for the first time, then be recalled in the discussion supported by the experimental results.

Minor Issues:

- The 8th reference indicating cost of WGS is not clear and accessible enough.
- The results start with validation of the choice of Tanzanian cohort and go on with validation of the suggested approach. Imputation accuracy improvement is presented with two measures: INFO score and r^2. Improvement in INFO score is illustrated in Table 1. The authors also mention increase in r^2; however, it is not illustrated in a concrete manner. R^2 is an ambiguous quantity per se, therefore it would be nice to support r^2 with numeric results too.
- Again, for an audience with other backgrounds than genetics, it's not quite clear from the beginning what *genome-wide fixation index* measures and how can we arrive to the

conclusions about genetic differentiation between populations. The authors could prefer providing a reminder about what this term is so that the reader won't have to look it up elsewhere. This way, the first section of the results could provide the necessary basics for the reader to follow the illustrated case easily.

- The discussion falls short of providing guidelines or at least ideas for a future work. This might be a matter of the authors' preference, but I wanted to put a pin in case they would like to revise.
