# Supplementary material for: Using population-specific add-on polymorphisms to improve genotype imputation in underrepresented populations
Source: PLoS Comput Biol. 2022 Jan 13;18(1):e1009628. doi: 10.1371/journal.pcbi.1009628 (PMC8791479; doi:10.1371/journal.pcbi.1009628)
Supplement: Attachment — Submitted filename: reponse_to_reviewers.pdf [file pcbi.1009628.s011.pdf]

GLOBAL HEALTH INSTITUTE
SCHOOL OF LIFE SCIENCES

Prof. Jacques Fellay

EPFL SV / GHI
Station nº19
CH-1015 Lausanne
Switzerland

Phone: +4121 693 18 49
Fax: +4121 693 72 20
E-mail: jacques.fellay@epfl.ch
Admin: marisa.marcianowynn@epfl.ch
Phone: +4121 693 72 32
Website: http://fellay-lab.epfl.ch

EPFL

September 2nd, 2021

Dear Editor,

Enclosed please find our revised manuscript entitled "**Using population-specific add-on polymorphisms to improve genotype imputation in underrepresented populations**" for your consideration.

We would like to thank all the reviewers for their insightful comments and suggestions. Their contributions have enabled us to greatly improve both the content and clarity of the manuscript.

In summary, we have conducted the following additional analyses to address some of the major issues raised:

1) We compared the baseline imputation performance of the Tanzanian cohort against populations that are represented in the AFGR reference panel. Since the Tanzanian population is genetically differentiated from these populations, we observed lower imputation performance as expected.

2) We compared the performance of add-on tag SNPs selected by the proposed method against those selected by random and by an existing software (Tagger). We showed that our proposed approach outperforms both methods.

Please see below point-to-point responses (with line numbers referring to the clean version of the manuscript).

Thank you again for your consideration of this manuscript.

Sincerely,

J. Fellay

Prof Jacques Fellay, MD, PhD

Reviewer #1:

The sub-optimal performance of genotyping array in populations that were not represented in the dataset used for its design is well-known. To address this challenge the authors have proposed an approach that would use whole-genome sequencing of representative samples from such a cohort (especially for under-represented populations) for the identification of tag SNPs that are valuable for capturing genetic diversity/LD of the population but are absent in the current genotyping array. Using a computationally improved array they further show that the inclusion of these addon tag SNPs could lead to better imputation and thereby increase the genomic coverage of the dataset. So while this study addresses a key genomic problem I have the following major concerns:

a. My first major concern is that while this approach is sound and effective in theory, the implementation is not straightforward and perhaps not even feasible in some scenarios. Firstly, the cost of sequencing ~100-150 high coverage whole genome sequences is still non-trivial, especially for many African and other under-representative populations. The same amount can be spent to genotype more individuals to increase power of the association study. Secondly, it might be extremely difficult to convince the array manufacturers to add on thousands of novel /continent-specific SNPs, especially if the cohort is small. Thirdly, not all SNPs work on all arrays genotyping platforms, so normally during array design, there is a bit of back and forth between what should go in and what is technically possible, this requires the active participation of both the research group and the manufacturer's bioinformatics team and is time and energy-consuming. Some platforms even need to experimentally validate the SNPs before adding them to the array which could further delay this. Thirdly, oftentimes these arrays are saturated in terms of bead pools so adding new bead pools might not be easy.

Response: We thank the reviewer for raising these thoughtful concerns, which we agree are indeed important to consider. While some issues raised are limitations of our proposed approach, many have already been directly addressed.

1) With regards to application in GWAS, the cost-effectiveness of genotyping more samples or to follow our proposed approach that relies on sequencing a small subset lies in a balance between power and sensitivity. We do envision that our proposed method would be more suitable for large cohorts: only a small subset would need to be sequenced under a fixed cost to improve sensitivity for the entire genotyped cohort, thus overall cost-effectiveness increases with sample size. We agree that for small cohorts, given a fixed cost, power gain from genotyping more individuals may be preferred over any sensitivity gain.

   We have clarified this in the Discussion section (Line 240 - 246).

2) As an example, Illumina is an array manufacturer that offers the possibility of customization through add-on content. An add-on of 5000 or 20,000 probes is available for the commercially available H3Africa array, with our design based on the 5000 probes add-on. However, indeed there is a minimum order requirement of 1152 samples.

   We have mentioned this in the Introduction section (Line 40 - 42) and addressed the limitation in the Discussion section (Line 245 - 248).

3) We agree that not all SNPs work on all genotyping arrays, and this is a valid concern that we have addressed in our proposed approach. In the case of the Illumina platform, the suitability of each SNP is publicly available to researchers, and no physical “back and forth” is required. The quality of probes that assay each SNP is predicted by a “probe-ability” score, which we use as one of the metrics to select the optimal add-on tag SNP. Specially, we excluded SNPs that have low assay success rates (probe-ability < 0.3), and incorporated the score in the ranking of candidate tag SNPs as well.

   We have clarified this in the Discussion section (Line 259 - 268).

4) No bead pools were saturated as a result of the 5000 probe add-on which we chose, but indeed this could become an issue with more add-ons. The proposed approach would then have to be modified to balance the saturation of existing bead pools against the suitability of the probe and the tagging efficiency of the tag SNP.

We have mentioned this as a limitation in the Discussion section (Line 268 - 272).

b. The improvement of imputation by addition of tag SNPs in the array is expected, so what assumes importance in this study is the extent of the improvement and also a demonstration that the specific method/algorithm that the authors use for SNP selection is leading to improvement beyond what would have been achieved by randomly adding common novel SNPs to the array. Also, comparisons to show that the method used has better (or least similar) performance in comparison to currently available approaches for tag SNP selection is critical.

Response: We thank the reviewer for the suggestion, which was suggested by Reviewer #2 as well.

In the updated manuscript, we demonstrate that add-on tag SNPs selected by our proposed approach do indeed outperform add-on tag SNPs selected by random and by an existing approach (Tagger software).

We show that the improvements in the imputation of target variants are more significant based on add-on tags derived from our proposed approach compared to those selected by random (Line 152 - 175; New Figure 3 and Figure 4). We also show that the efficiency of add-on tags selected by our proposed approach outperform those selected by random and selected by Tagger (Line 176 - 211; Table 1), especially when considering the per probe efficiency rather than per tag efficiency in the case of Tagger.

I would recommend that the authors rewrite this paper focusing on their approach for tag SNP selection and comparing its performance to competing methods.

Response: Since we have addressed issues raised in comment a. regarding feasibility, we believe our proposed approach would still be of interest to researchers in terms of addressing the need to improve genotype imputation (and consequently the sensitivity of association studies) in underrepresented populations.

Reviewer #2:

In the manuscript "Using population-specific add-on polymorphisms to improve genotype imputation in underrepresented populations" by Xu et al., the authors described a heuristic in which they leverage the WGS data from a small subset of the population of interest to identify variants that are not well-imputed, and select additional SNPs to improve the coverage through imputation. A disparity exists when the study population is not represented in external genomic references (such as the 1000 Genomes), and this framework would help alleviate the situation. Thus, in general even though the framework described is a heuristic, there are both interests and value in the described work. I have the following comments, some more major than others.

Major comments:

1. It would be better if the authors can describe the specific situation they envisioned for the application of their framework. As described, the heuristic requires a subset of individuals first be already genotyped using the H3Africa Array, and then identify the add-on content. Are the authors envisioning that this approach will identify add-on content, which will then be added to the H3Africa Array as customized content to genotype the rest of the cohort? Does H3Africa Array actually allow customized content? How much content is allowed? For readers who are not familiar with the design of genotyping arrays it would be important to introduce this concept early in the Introduction.

   Response: We apologize for not being clear about this in the original manuscript.

   Our framework relies on the whole-genome sequencing (WGS) of a subset of a cohort for two purposes: 1) To inform the selection of population-specific add-on tag SNPs, such that the rest of the cohort could be array-genotyped with the improved array, and 2) To construct an internal population-specific reference panel that supplements external publicly available reference panels.

   For the presented proof-of-concept example based on the Tanzanian cohort (TB-DAR), we did indeed design add-on content for the H3Africa array. Illumina, which manufactures the array, offers the option of a 5000 or 20,000 probe add-on. We chose the 5000 probe add-on for the study.

   We have updated the Introduction section (Line 39 - 69) to include discussions regarding the objective of the WGS subset, information regarding the H3Africa array, and scenarios that we envision our framework to function.

2. In practice, researchers are often faced with availability of an extremely large imputation reference (i.e. the TOPMED panel) in which the specific population being studied (e.g. the Tanzanians) is not represented. But by including the add-on content, is the intention that the genotyped cohort will be imputed with the large reference (TOPMED) or the small population-specific reference (Tanzanian), or some form of cosmopolitan approach? If the former, the current heuristic would be restricted to identifying add-on SNPs that already exist in the large reference (TOPMED), and would only work for target SNPs that also already exist in the reference. If the latter, a researcher would then obviously lose the much greater spectrum of variation that exists in the large reference. In the Discussion the authors only mentioned how population-specific variants from Tanzania would be missed, but I think the general drawback that variation discovered in the population-specific WGS are under-utilized should be discussed in greater length.

   Response: Again, we apologize for not being clear about this in the original manuscript.

   Our approach was for the genotyped cohort to be imputed based on a cosmopolitan approach. Specifically, we utilized both the AFGR reference panel (the largest publicly available reference panel for the African populations) and an internal Tanzanian reference panel constructed based on a training set derived from the WGS data. For each site, we derived the genotype call based on the reference panel with higher predicted imputation accuracy for the site. We have clarified this in the Introduction (Line 85 - 88) and Methods (Line 373 - 384).

While population-specific variants from Tanzania were not explicitly targeted, this does not mean that they would be missed. We explicitly targeted poorly imputed SNPs, and one (but not the only) reason that an SNP may be poorly imputed is because it is specific to the Tanzania population and thus not represented in the existing array design. We have clarified this in the Discussion section (Line 249 - 258)

3. In the specific example of Tanzanian in this manuscript, the authors spent the first section of Results (Figure 3) to show that the Tanzanian population is differentiated from other African populations included in the 1000 Genomes. However, the authors did not actually demonstrate that imputation quality is impacted given the differentiation. What would happen if you just impute Tanzanian population using the existing imputation reference? Is it actually significantly worse than imputation for other African populations (such as the West Africans that have closer imputation reference)? Demonstrating this would better motivate the need for the framework developed here.

   Response: We thank the reviewer for the suggestion. We have included additional analyses which demonstrate that as expected, given the differentiation between the Tanzanian population (TB-DAR) and populations represented in the AFGR reference panel, imputation performance is lower in the Tanzanian population compared to those already represented. Specifically, we show that based on the AFGR reference panel, the fraction of sites successfully imputed in the Tanzanian cohort is lower than the 1000 Genomes populations that are represented in the reference panel.

   Please see the updated results section for details (Line 127 - 137, New Figure S3).

4. The comparisons in Figure 4 are also a bit misleading. The target SNPs were chosen to be poorly imputed in the first place, and by adding more SNPs in the region by definition there will be an improvement in imputation quality (at worse, the imputation quality would not change). The authors would need to show that there is an improvement over just randomly adding more SNPs to improve the imputation.

   Response: We thank the reviewer for the suggestion, which was suggested by Reviewer #1 as well.

   In the updated manuscript, we demonstrate that add-on tag SNPs selected by our proposed approach do indeed outperform add-on tag SNPs selected by random.

   We show that the improvements in the imputation of target variants are higher based on add-on tag SNPs derived from our proposed approach compared to those selected by random (Line 152 - 175; New Figure 3 and Figure 4). We also show that the efficiency of add-on tags selected by our proposed approach outperform those selected by random (Line 176 – 211: Table 1).

5. The current framework uses only single-marker tagging to identify add-on content. The authors should be able to improve efficiency further by considering multi-marker tagging. Can the authors comment on this possibility in the manuscript?

   Response: We have commented on the possibility regarding the use of multi-marker tagging to improve the efficiency of the add-on tags.

   This has been updated in the discussion section (Line 294 - 296)

6. Finally, it would be helpful if the authors can explain certain figures in more detail. For example, Figure S4 seems to be an important illustrative example of the algorithm, but the authors provided no substantial explanation in the Methods or the Figure Legend for the readers to follow and/or appreciate the figure. Furthermore, Figure 1 is referenced only in passing in the Introduction, with little explanation. The authors should explain / utilize the figure more extensively, or perhaps just drop Figure 1 from the manuscript.

   Response: For the old Figure S4 (new Figure S6), we have added a detailed step-by-step description in the Methods section (Line 407 - 428)

For the old Figure 1 (new Figure S1), we have added an explanation in the Introduction section (Line 54 - 69)

Minor comments:

1. Line 17: reference 6 (the Sardinia paper) seems to be inappropriately cited here. WGS was generated in this paper, but used as imputation reference for the remaining individuals with only genotype data. This is a closer use case as described by the proposed framework in this manuscript, rather than a full WGS for association testing.

   Response: We thank the reviewer for noticing this, this was a mistake on our part. We have updated the reference to refer to a fully WGS based GWAS, conducted in the Qatari population (Thareja et al, 2021: https://www.nature.com/articles/s41467-021-21381-3?proof=t). This is reference 7 in the updated manuscript.

2. Figure 3A: the Fst values are rather small. The largest value is about 0.01 – this is on the order of differentiation among European populations, though I would have expected the differentiation to be greater among different African populations (see https://science.sciencemag.org/content/367/6484/eaay5012.long). Can the author explain what might lead to the lower-than-expected Fst among African populations?

   Response: We thank the reviewer for the insightful comment. The differentiation is indeed larger among different African populations, with the African Genome Variation Project (AGVP) suggesting a mean Fst of 0.027 between East and West African populations (Gurdasani et al, 2014: https://www.nature.com/articles/nature13997 , see Supplementary Table 1). The maximum value we report (between LWK and GWD, Fst = 0.011) is an underestimate since we only compared 1000 Genomes population, which by no means represent the full extent of genetic diversity of Sub-Saharan African populations. The values we calculate for the 1000 Genomes populations are nevertheless very similar to those found in existing studies (for example, Cao et al, 2020: https://www.nature.com/articles/s41422-020-0322-9/figures/4).

   We have clarified this in the Results section (Line 120 - 124)

3. Line 236: was there any one actually excluded due to relatedness?

   Response: A single individual was excluded due to relatedness in the Tanzanian (TB-DAR) cohort, and 281 in the 1000 Genomes Sub-Saharan African populations.

   We have updated the methods section (Line 355 - 359)

4. Line 238: Long-range LD regions were excluded when performing PCA, but the long-range LD is based on European populations. Is it appropriate to exclude them in African-based PCA?

   Response: We agree that since the pre-defined Long-range LD regions were based on European populations, they may not be portable to the African populations. Thus, we have re-defined long-range LD regions in the African populations that we considered using a clustering algorithm proposed by Privé et al (https://academic.oup.com/bioinformatics/article/34/16/2781/4956666). We excluded these regions as before. See Methods (Line 345 - 347) for details.

   We confirmed that PCs are capturing population structure and not Long-range LD regions by checking the PC loadings of each SNP across the genome. The figure below shows that after pruning and removal of Long-range LD regions, PC loadings are uniformly distributed. This suggests the absence of any remaining Long-range LD regions that affect PC1 – PC4.

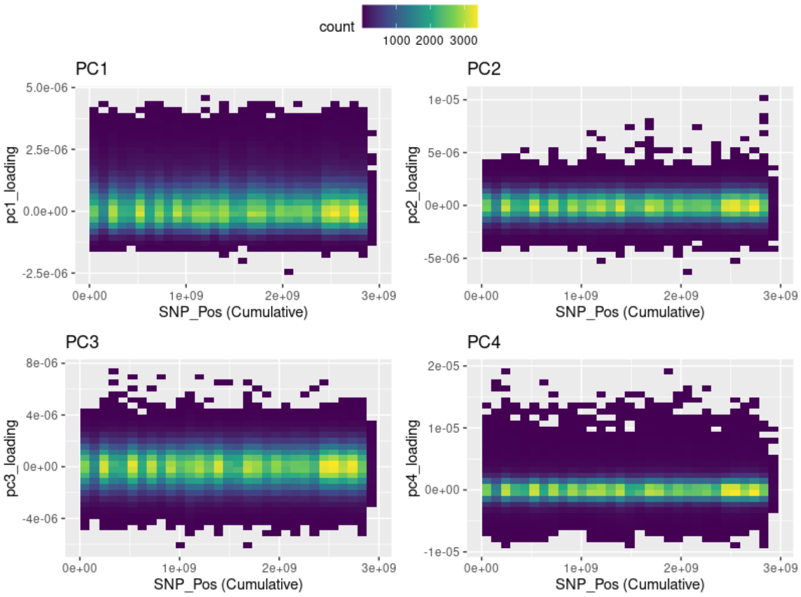

As such, the results of the PCA analysis remained very similar to the original analysis.

5. Line 298: this is specific to Illumina platform, I assume. Please state this explicitly and explain why it is a different value for A/T or C/G SNPs vs. all other SNPs.

Response: Yes, this is specific to the Illumina platform. We have clarified this in the Results section (Line 202 - 204).

6. Line 318: can you better define "spread"?

Response: For example, the spread of well-imputed variants is defined as $\frac{\sigma_{INFO>0.8}}{\max(\sigma_{INFO>0}, \sigma_{WGS})}$ where σ represents the standard deviation of the positions of SNPs under each criterion (imputed with INFO > 0.8, all imputed SNPs, and all sequenced SNPs)

We have added this in the Methods section (Line 442 - 446).

Reviewer #3:

The manuscript proposes an approach to increase imputation efficiency on SNP array data from populations poorly represented in public databases. The method consists of submitting a subset of the population of interest to WGS, and then use this information to select a more suitable set of population-specific SNPs to increase the quality of imputation. Their method show to be usefull and can be conveniently applied mainly to data from isolated populations. There are some minor issues outlined below that I recommend to be addressed.

1- Throughout the text (for example in the Figure 1 legend) the authors refers to common and rare SNPs. A genetic polimorphism is a phenomenom related to the allele frequencies of a single genetic locus and can not be rare or common when a single population is considered. They are referring to rare and common alleles it would be better to correct it, making the text more precise and clear. In addition, when the alternative allele of a genetic marker is too rare, by definition it could not even be classified as an SNP.

Response: We apologize for the ambiguity here. By referring to an SNP being rare or common, we intended to refer to its minor allele frequency (MAF) in the study population. It is indeed possible to confuse this with the population-specificity of a SNP (i.e. common referring to the alternative allele being present in high frequency in multiple populations, and rare referring to the alternative allele being present in high frequency in only a few populations). We are also aware of the definition of SNPs as being single-nucleotide substitutions that are present above a 1% frequency.

In the updated manuscript, instead of common/rare SNPs, we refer to common/rare variants. Furthermore, we specify the population(s) to which the frequency refers to.

2- The authors present the add-on SNPs count per chromosome obtained from Setting 1 and Setting 2. Why the pattern of the distribution observed in the barplot of add-on SNPs is so different from that observed for the tag SNPs of the H3Africa array? Wouldn't be expected that longer chromosomes have more add-on SNPs than shorter ones? Besides, Fig. S2-A (Setting 1) shows that the count for chromosome 6 is much higher than the counts obtained from all other autosomal chromosomes. Why? It is known that chromosome 6 includes the MHC region, where genotype determination may present read mapping dificulties related to the short reads generated by high-throughput sequencing. Potential confounding factors for the reliability of MHC region are genotyping the extent of sequence level, structural polymorphism, and the choice of reference sequence. Has any extra care been taken regarding the variant calling of this specific region? Could the MHC region be inflating the chromosome 6 count of Setting 1? Could this be a source of bias?

Response: We thank the reviewer for the thoughtful comments. The distribution of add-on SNPs is dependent on the precise regions for which imputation is of poor quality, since it was only in such regions where add-on tag SNPs were selected under Setting 1. This does not necessarily need to be correlated with chromosome length, given that poorly imputed regions do not necessarily have to be uniformly distributed across the genome. On the other hand, if even coverage was the selection paradigm used by the array design, then tag SNPs of the H3Africa array would be expected to be uniformly distributed across the genome and thus expected to be correlated with chromosome length. Qualitatively, this is indeed what is observed in Figure S2-B (New Figure S4-B).

Indeed, the add-on tags within the MHC region do contribute strongly to the overall high count of chromosome 6 under Setting 1, since the MHC region was purposefully selected under Setting 1 as a region of interest (due to its involvement in immunity and contribution to infectious disease prognoses). Furthermore, due to the highly polymorphic nature of the region, we relaxed the MAF threshold (0.01 instead of 0.05) for a poorly imputed variant to be considered as a target variant. Since the MHC region was explicitly targeted to ensure high coverage, we do not believe this could be characterized as a source of bias.

Nevertheless, we agree that variant calling could be improved in the region, through the use of alternative contigs of the reference genome. Furthermore, efficiency could be improved by selecting tag SNPs that directly target HLA alleles rather than all common variation.

We have commented on these limitations in the Discussion section (Line 288 - 294)

3- Any missingness data cleaning was performed across markers? Or only at individual level? It is recommended to remove markers with high levels of missing data.

Response: Only SNPs with missingness below 0.5 were included as candidate tag SNPs. (Line 397)

4- Regarding the estimation of within population differentiation, the method used by the authors, although creative, seems to be biased and incorrect. For two main reasons:
(1) The two top principal components of tha PCA acounts for only a small fraction of the total variation (less than 2% for data shown in FigS1);
(2) If the researchers have no information about the existence of population substructure this estimate makes no sense. Otherwise they would have to attribute each individual in the corresponding subpopulation and then estimate Weir & Cockerham pairwise Fst; and this procedure would be correct only if the population is subdivided in exactly two subpopulations. If the researchers have no clue about substructuring and want to check it, the proper way consists in estimate original Wright's fixation index Fst = var(p)/[p(1-p)], which will consider populations with any number of subpopulations.
Since the authors have no reason to believe that there is a substructure in any population, I recommend the exclusion of this analysis or repeating the estimate in a more appropriate way.

Response: We thank the reviewer for the suggestion. We agree that the procedure we used to estimate within-population Fst was not optimal, as it relied on the assumption that there exist only two subpopulations within a population. We agree that the PCA analysis indicates the absence of significant sub-structure within each population, and thus excluded this analysis as suggested.

Nevertheless, we did aim to address this in the updated manuscript. We now use the Hudson’s estimator instead of the Weir and Cockerham (WC) estimate for pairwise Fst. It has been shown that when Fst is not identical for both populations, the WC estimate is biased according to the ratio of sample sizes of the two populations (Bhatia et al, 2013: https://genome.cshlp.org/content/23/9/1514). The Hudson’s estimate is unbiased under such a scenario.

Results obtained with Hudson’s estimate were very similar to our previous analysis, with the interpretation unchanged (See New Figure 2B).

Reviewer #4:

Review Summary:

Using population-specific add-on polymorphisms to improve genotype imputation in underrepresented populations

In Genome-Wide Association Studies (GWAS), statistical inference of untyped variants in genome is needed to enhance coverage of genotyping arrays, which is referred as genotype imputation. The imputation process is performed based on external reference panels where observed correlations between variants on these panels can be indicative of untyped variants on the studied panels. The more genetically similar the reference and study populations are, the more reliable the process of genotype imputation becomes. Here, the problem lies with underrepresented populations, for whom, the reference panels on hand may not be adequately representative due to their population-specific genetic structures. In such cases, selected SNPs through genotype imputation relying on existing reference population may fall short of the actual genetic variation in the study population.

As a remedy, Whole-Genome Sequencing (WGS) can be suggested to carry out GWAS in underrepresented populations. Although, necessity of large sample sizes compromises high- priced whole-genome sequencing despite its potential. Alternately, population-specific reference panels can be constructed to improve genotype imputation. However, such efforts still remain insufficient to capture the entire genetic variability in African populations notably, due to relatively higher genetic diversity among them. Accordingly, the authors propose a cost-effective solution to the poor genotype imputation issue, that involves selection of population- specific SNPs to add to available genotyping arrays. WGS of 10% of the entire sample can benefit the inclusiveness of available reference arrays. Also, WGS of sub-sample reveals population-specific allele frequency and haplotype structure differences, that can help the selection of add-on SNPs, thus enhancing the imputation of poorly tagged SNPs in underrepresented populations. Furthermore, the approach is claimed to improve haplogroup calling in mitochondria and Y chromosome variants.

The authors showcase their approach on a Tanzanian cohort, which is not incorporated in the existing reference panels. They validate their choice of cohort by computing the genetic difference between the selected Tanzanian cohort and 1000 Genomes African population with respect to genome-wide fixation index. Here, the usefulness of the population-specific add-on SNPs for Tanzanian cohort seems justified. This comparison provides a ground to conclude that geography play a role in genetic differentiation of African populations such that geographically closer populations have more genetic similarities, which is also supported by a genetic principal component analysis.

Selection of add-on SNPs is performed under two settings with different objectives: One guaranteeing coverage and the other, efficiency. For evaluation of the approach, imputation quality is measured on the priorly divided WGS test sample, while selected add-on SNPs under both settings are included separately while tagging target SNPs. Under both settings, an overall improvement of imputation accuracy in terms of INFO score and r2 is obtained. The authors illustrate these findings with an exemplary region, where they observe proximity between add- on and poorly tagged target SNPs. Finally, mitochondria and Y chromosome haplogroups are analyzed in the cohort where a certain number of haplogroup marker SNPs are selected as add- ons. Here, the accuracy of haplogroup calling is increased by 22% in case of mitochondrial variants compared to the existing reference array, whereas no such improvement is gained for Y chromosome possibly due to sufficient coverage of the existing reference array. In the end, experimental results on the Tanzanian cohort affirm that consideration of poorly imputed add- on SNPs on the existing reference arrays improves genotype imputation of the common variants in the underrepresented cohort.

Comments:

First of all, I really enjoyed reading this paper. The style is easy to follow and the authors accompany the reader all the way for a better understanding of the topic.

The introduction successfully connects the sources of the problem of improving imputation to a clear statement of the purpose of this work. The potentials and drawbacks of available remedies to the issue are clearly stated which pave the way for the suitability of the suggested study. Choice of Tanzanian cohort is reasoned properly. The

authors are able to voice the strengths of their approach in terms of its generalizability to any other population, its cost- effectiveness and promise in empowering GWAS. I would like to congratulate them for their work.

Some major and minor issues I might suggest revising are given below:

Major Issues:

- In the introduction, the potentials and drawbacks of available remedies to the issue are clearly stated which pave the way for the suitability of the suggested study. However, how the authors deduce insufficient coverage of African genetic variation from the references about relatively high genetic diversity of African Populations remains rather questionable and unsupported by references. It would be really nice to strengthen the motivation of the approach at this point.

  Response: We apologize for not being clear about this. We meant to convey that given that the current publicly available reference panels only represent a subset of African populations, there exist populations where imputation is still suboptimal due to underrepresentation. This is exacerbated by the fact that there is higher genetic diversity across African populations. This means that compared to other populations, reference panels would need to be even larger and more diverse to capture the full extent of genetic diversity and thus achieve a similar level of imputation accuracy.

  We have clarified this in the Introduction section (Line 28 - 35).

- An improvement of 22% in haplogroup calling in mitochondria is stated in the final section of the results, whereas no such improvement is observed in the case of Y chromosome, which is said to be caused by lack of add-on SNPs. But the last phrase in the introduction indicates a useful selection scheme for mitochondria and Y chromosome, when in fact such a selection scheme did not work for Y chromosome. The results could be better laid out if the authors would tone down their claims for Y chromosome in the introduction.

  Response: We thank the reviewer for the comment. We have updated the Introduction section to improve clarity with regards to Y chromosome haplogroup calling, stating that existing H3Africa array content was mostly sufficient to achieve accurate Y chromosome haplogroup calling. (Line 89 - 91)

- The first paragraph in discussion refers to previous studies implementing the idea proposed in this paper, both for Type 2 Diabetes, and states their outcomes as a supporting evidence. Moving this statement to the introduction in the related work might make more sense so that the reader is exposed to all the related work and current state- of-the-art from the beginning. The contribution of this paper on top of these previous applications should also be stated in the introduction for the first time, then be recalled in the discussion supported by the experimental results.

  Response: We thank the reviewer for the suggestion. In the Introduction section, we have added a summary of previous work regarding the use of internal panels as suggested. (Line 45 - 53)

Minor Issues:

- The 8th reference indicating cost of WGS is not clear and accessible enough.

  Response: We thank the reviewer for spotting this, we have added an url to this reference.

- The results start with validation of the choice of Tanzanian cohort and go on with validation of the suggested approach. Imputation accuracy improvement is presented with two measures: INFO score and r2. Improvement in INFO score is illustrated in Table 1. The authors also mention increase in r2; however, it is not illustrated in a concrete manner. R2 is an ambiguous quantity per se, therefore it would be nice to support r2 with numeric results too.

Response: We have included the r2 measure when comparing the improvement in imputation performance of target variants (New Figure 3 and Figure 4), but did not include r2 when comparing improvement in imputation performance of all imputed variants within the same regions (Table 1). Since r2 measures the concordance between the imputed genotypes and ground truth genotypes within the relatively small testing set (N = 29), the measure could only be accurately derived for common target variants in the study population (MAF > 0.05). We excluded r2 in Table 1 since r2 cannot be derived for all imputed variants (including those not explicitly targeted), while INFO score can be derived.

- Again, for an audience with other backgrounds than genetics, it's not quite clear from the beginning what *genome-wide fixation index* measures and how can we arrive to the conclusions about genetic differentiation between populations. The authors could prefer providing a reminder about what this term is so that the reader won't have to look it up elsewhere. This way, the first section of the results could provide the necessary basics for the reader to follow the illustrated case easily.

  Response: We thank the reviewer for the suggestion. We have added a description of Fst in the Results section (Line 103 - 106)

- The discussion falls short of providing guidelines or at least ideas for a future work. This might be a matter of the authors' preference, but I wanted to put a pin in case they would like to revise.

  Response: We have added ideas for future work (Improvements in MHC region, multi-marker tagging) in the Discussion section (Line 288 - 295)
