# Supplementary material for: Using population-specific add-on polymorphisms to improve genotype imputation in underrepresented populations
Source: PLoS Comput Biol. 2022 Jan 13;18(1):e1009628. doi: 10.1371/journal.pcbi.1009628 (PMC8791479; doi:10.1371/journal.pcbi.1009628)
Supplement: Attachment — Submitted filename: PLOS Review - Revised Version .pdf [file pcbi.1009628.s012.pdf]

Revised Version:

Using population-specific add-on polymorphisms to improve genotype imputation in underrepresented populations

Comments:

I have pointed out the following major and minor issues I noticed while reading this paper for the first time. Having read the revised version, I would like to start with congratulating the authors for their efforts and their clarity in addressing all the revision issues.

I would like to respond all the updates in the paper under the relevant issues previously stated by myself below:

Major Issues:

- *In the introduction, the potentials and drawbacks of available remedies to the issue are clearly stated which pave the way for the suitability of the suggested study. However, how the authors deduce insufficient coverage of African genetic variation from the references about relatively high genetic diversity of African Populations remains rather questionable and unsupported by references. It would be really nice to strengthen the motivation of the approach at this point.*

  In the reviewed version, the authors address the ambiguity by motivating the need for more extensive imputation reference panels thanks to a clear comparison with non-African populations. The introduction enunciates now why one would deem the coverage of African genetic diversity insufficient, which relates more naturally to the actual goal of this work.

- *An improvement of 22% in haplogroup calling in mitochondria is stated in the final section of the results, whereas no such improvement is observed in the case of Y chromosome, which is said to be caused by lack of add-on SNPs. But the last phrase in the introduction indicates a useful selection scheme for mitochondria and Y chromosome, when in fact such a selection scheme did not work for Y chromosome. The results could be better laid out if the authors would tone down their claims for Y chromosome in the introduction.*

The claims in the introduction about Y chromosome reads now in line with the results section, such that the authors add that the coverage of Y chromosome is sufficient for haplogroup calling. I believe this addition should be helpful for a first-time reader to follow the results section that complies with the expectations raised in introduction.

- *The first paragraph in discussion refers to previous studies implementing the idea proposed in this paper, both for Type 2 Diabetes, and states their outcomes as a supporting evidence. Moving this statement to the introduction in the related work might make more sense so that the reader is exposed to all the related work and current state-of-the-art from the beginning. The contribution of this paper on top of these previous applications should also be stated in the introduction for the first time, then be recalled in the discussion supported by the experimental results.*

  Inclusion of previous work helped the introduction section to read more thoroughly, as it seemed rather late to read about other studies that applies the suggested approach to different problems. Although, my understanding from the revised version of the paper, between the lines 45-53, is that the authors lay out the previous work as the second reasoning for their approach. Such a narrative might complicate the main theme in the paragraph, even leave the bridge to the contribution of the paper up in the air. While the revision about this issue is really on point, it would be even nicer if the authors reorganize or reformulate the paragraph with clearer purposes.

Minor Issues:

- *The 8th reference indicating cost of WGS is not clear and accessible enough.*

  The reference has now an URL and it is accessible.

- *The results start with validation of the choice of Tanzanian cohort and go on with validation of the suggested approach. Imputation accuracy improvement is presented with two measures: INFO score and* r^2*. Improvement in INFO score is illustrated in*

Table 1. The authors also mention increase in r^2; however, it is not illustrated in a concrete manner. R^2 is an ambiguous quantity per se, therefore it would be nice to support r^2 with numeric results too.

The authors chose to include r^2 for common target variants in the study population and explained clearly how it's the only appropriate comparison to present this measure. When it comes to improvement in imputation performance of all imputed variants within the same regions, the authors did not include r^2 in Table 1 since it cannot be derived for all imputed variants. Having read the reasoning, I agree with the authors' choice and thank them for their explanations about the issue.

- *Again, for an audience with other backgrounds than genetics, it's not quite clear from the beginning what genome-wide fixation index measures and how can we arrive to the conclusions about genetic differentiation between populations. The authors could prefer providing a reminder about what this term is so that the reader won't have to look it up elsewhere. This way, the first section of the results could provide the necessary basics for the reader to follow the illustrated case easily.*

The authors have added a quite concise definition of the genome-wide fixation index which also includes what the extreme values in the range 0-1 represents for the metric. I would like to thank them for this update.

- *The discussion falls short of providing guidelines or at least ideas for a future work. This might be a matter of the authors' preference, but I wanted to put a pin in case they would like to revise.*

My final point was rather a suggestion than an issue, but it has been really nice that the authors agreed with it. The paper now raises ideas and even guides the way for further research, especially based on the limitations faced in this work such as the need for more accurate variant calling in MHC regions, or multi-marker tagging instead of single-marker tagging. I find the revision as regards to this suggestion has been carried out successfully.
