# Supplementary material for: Using population-specific add-on polymorphisms to improve genotype imputation in underrepresented populations
Source: PLoS Comput Biol. 2022 Jan 13;18(1):e1009628. doi: 10.1371/journal.pcbi.1009628 (PMC8791479; doi:10.1371/journal.pcbi.1009628)
Supplement: Attachment — Submitted filename: reponse_to_reviewers.pdf [file pcbi.1009628.s013.pdf]

GLOBAL HEALTH INSTITUTE
SCHOOL OF LIFE SCIENCES

Prof. Jacques Fellay

EPFL SV / GHI
Station nº19
CH-1015 Lausanne
Switzerland

Phone: +4121 693 18 49
Fax: +4121 693 72 20
E-mail: jacques.fellay@epfl.ch
Admin: marisa.marcianowynn@epfl.ch
Phone: +4121 693 72 32
Website: http://fellay-lab.epfl.ch

November 2nd, 2021

Dear Editor,

Enclosed please find our revised manuscript entitled "**Using population-specific add-on polymorphisms to improve genotype imputation in underrepresented populations**" for your consideration.

We would like to again thank all the reviewers for their comments during this second round of revision.

Please see below point-to-point responses to remaining concerns raised by reviewer 1 and reviewer 2.

We have also modified the Data Availability statement to address that individual level data cannot be made publicly available due to privacy related restrictions, but added a link to where summary level statistics are stored.

Thank you again for your consideration of this manuscript.

Sincerely,

Prof Jacques Fellay, MD, PhD

Reviewer #1:

The authors have addressed most of the main concerns. There in one aspect I would like to comment on. As some of the variable such as admin/MTA, logistics, time delays (probably in the order of 3-6 months -including shipping, sequencing, selection of additional variants based on WGS and their addition to array), which are also major consideration have not been accounted for in this study. I would suggest the authors to add a couple of lines in the discussion to clarify this..

We would like to thank the reviewer for this comment. We agree with the reviewer that our proposed approach can be time-consuming to implement due the reasons listed. Thus, those interested should first check whether their study population of interest is well-represented in existing array designs and reference panels, and then consider whether the sensitivity gain from the proposed approach may be worthwhile. We have updated the discussion section to clarify this (Line 277 - 283).

Reviewer #2:

I'd like to thank the author for addressing my concerns and implementing new analyses based on my previous review. I think the manuscript is now much clearer. I have some additional comments regarding the current revised manuscript and new analyses:

We would like to thank the reviewer for the additional comments.

1. Line 45, the author mentioned the strategy to supplement external reference panels with WGS samples from an internal study cohort. This is possible perhaps in the particular scenario with the AFGR panel. However, this is increasingly impossible – given the sample size of external panels such as TOPMED. It is worth to acknowledge this possibility at some point in the manuscript. In fact, what is lacking in the current manuscript is an actual comparison that AFGR, with or without the internal Tanzanian reference, would outperform the default approach of just using the TOPMED reference panel. It would be good to show that using a smaller AFGR actually is an improvement over TOPMED, and then the authors' heuristic will further improve upon that.

We chose the AFGR reference panel as it was the reference panel with the highest representation of African populations at the conception of our study (September 2019). To clarify our choice of the AFGR reference panel, we have included a comparison of the imputation performance of all relevant reference panels publicly available at that time point (See Results - Performance of external reference panels). We show that the AFGR outperforms the two other options.

We agree that with the release of larger and more diverse reference panels (e.g. TOPMED), the expected gain from the proposed approach is expected to decrease for many populations. However, until reference panels and corresponding array designs are able to capture almost all of the human genetic diversity, we still believe that our approach fills an important gap for certain underrepresented populations. We have clarified this point in the discussion section (Line 276 - 286).

2. The comparison to tagger shows that the benefit appears to be modest, if any (Table 1). Can there by confidence interval placed on these percentages such that a comparison between Tagger vs. Proposed Approach can be more directly evaluated?

We have added error bars to Figure 2 and confidence intervals to Table 1 (New Table 2) to illustrate the heterogeneity of imputation performance in each MAF bin and across genomic regions respectively. We also have re-formulated the results section (Line 210 - 213) to clarify the interpretation.

3. At times the writing become too colloquial – for example, the use of "array population" can be confusing, esp. with the similar use of a "study population". Perhaps the authors can directly define them upfront in text if the only use of these terms are only for convenience.

We have clarified what "array population" and "study population" refers to (See Introduction, Line 54-58)
