# Supplementary material for: Using population-specific add-on polymorphisms to improve genotype imputation in underrepresented populations
Source: PLoS Comput Biol. 2022 Jan 13;18(1):e1009628. doi: 10.1371/journal.pcbi.1009628 (PMC8791479; doi:10.1371/journal.pcbi.1009628)
Supplement: Attachment — Submitted filename: XuEtAl_PLoSCOMPBIO_2021.docx [file pcbi.1009628.s009.docx]

In the manuscript “Using population-specific add-on polymorphisms to improve genotype imputation in underrepresented populations” by Xu et al., the authors described a heuristic in which they leverage the WGS data from a small subset of the population of interest to identify variants that are not well-imputed, and select additional SNPs to improve the coverage through imputation. A disparity exists when the study population is not represented in external genomic references (such as the 1000 Genomes), and this framework would help alleviate the situation. Thus, in general even though the framework described is a heuristic, there are both interests and value in the described work. I have the following comments, some more major than others.

Major comments:

1. It would be better if the authors can describe the specific situation they envisioned for the application of their framework. As described, the heuristic requires a subset of individuals first be already genotyped using the H3Africa Array, and then identify the add-on content. Are the authors envisioning that this approach will identify add-on content, which will then be added to the H3Africa Array as customized content to genotype the rest of the cohort? Does H3Africa Array actually allow customized content? How much content is allowed? For readers who are not familiar with the design of genotyping arrays it would be important to introduce this concept early in the Introduction.
2. In practice, researchers are often faced with availability of an extremely large imputation reference (i.e. the TOPMED panel) in which the specific population being studied (e.g. the Tanzanians) is not represented. But by including the add-on content, is the intention that the genotyped cohort will be imputed with the large reference (TOPMED) or the small population-specific reference (Tanzanian), or some form of cosmopolitan approach? If the former, the current heuristic would be restricted to identifying add-on SNPs that already exist in the large reference (TOPMED), and would only work for target SNPs that also already exist in the reference. If the latter, a researcher would then obviously lose the much greater spectrum of variation that exists in the large reference. In the Discussion the authors only mentioned how population-specific variants from Tanzania would be missed, but I think the general drawback that variation discovered in the population-specific WGS are under-utilized should be discussed in greater length.
3. In the specific example of Tanzanian in this manuscript, the authors spent the first section of Results (Figure 3) to show that the Tanzanian population is differentiated from other African populations included in the 1000 Genomes. However, the authors did not actually demonstrate that imputation quality is impacted given the differentiation. What would happen if you just impute Tanzanian population using the existing imputation reference? Is it actually significantly worse than imputation for other African populations (such as the West Africans that have closer imputation reference)? Demonstrating this would better motivate the need for the framework developed here.
4. The comparisons in Figure 4 are also a bit misleading. The target SNPs were chosen to be poorly imputed in the first place, and by adding more SNPs in the region by definition there will be an improvement in imputation quality (at worse, the imputation quality would not change). The authors would need to show that there is an improvement over just randomly adding more SNPs to improve the imputation.
5. The current framework uses only single-marker tagging to identify add-on content. The authors should be able to improve efficiency further by considering multi-marker tagging. Can the authors comment on this possibility in the manuscript?
6. Finally, it would be helpful if the authors can explain certain figures in more detail. For example, Figure S4 seems to be an important illustrative example of the algorithm, but the authors provided no substantial explanation in the Methods or the Figure Legend for the readers to follow and/or appreciate the figure. Furthermore, Figure 1 is referenced only in passing in the Introduction, with little explanation. The authors should explain / utilize the figure more extensively, or perhaps just drop Figure 1 from the manuscript.

Minor comments:

1. Line 17: reference 6 (the Sardinia paper) seems to be inappropriately cited here. WGS was generated in this paper, but used as imputation reference for the remaining individuals with only genotype data. This is a closer use case as described by the proposed framework in this manuscript, rather than a full WGS for association testing.
2. Figure 3A: the Fst values are rather small. The largest value is about 0.01 – this is on the order of differentiation among European populations, though I would have expected the differentiation to be greater among different African populations (see <https://science.sciencemag.org/content/367/6484/eaay5012.long>). Can the author explain what might lead to the lower-than-expected Fst among African populations?
3. Line 236: was there any one actually excluded due to relatedness?
4. Line 238: Long-range LD regions were excluded when performing PCA, but the long-range LD is based on European populations. Is it appropriate to exclude them in African-based PCA?
5. Line 298: this is specific to Illumina platform, I assume. Please state this explicitly and explain why it is a different value for A/T or C/G SNPs vs. all other SNPs.
6. Line 318: can you better define “spread”?
